# Assessment of *Plasmodium falciparum* anti-malarial drug resistance markers in *pfk13*-propeller, *pfcrt* and *pfmdr1* genes in isolates from treatment failure patients in Democratic Republic of Congo, 2018–2019

**DOI:** 10.1186/s12936-021-03636-y

**Published:** 2021-03-11

**Authors:** Doudou M. Yobi, Nadine K. Kayiba, Dieudonné M. Mvumbi, Raphael Boreux, Pius Z. Kabututu, Hippolyte N. T. Situakibanza, Solange E. Umesumbu, Patrick De Mol, Niko Speybroeck, Georges L. Mvumbi, Marie-Pierre Hayette

**Affiliations:** 1 Department of Basic Sciences, Faculty of Medicine, University of Kinshasa, Kinshasa, Democratic Republic of Congo; 2 School of Public Health and Research Institute of Health and Society, Catholic University of Louvain, 1200 Brussels, Belgium; 3 School of Public Health, Faculty of Medicine, University of Kinshasa, Kinshasa, Democratic Republic of Congo; 4 Department of Public Health, Faculty of Medicine, University of Mbujimayi, Mbujimayi, Democratic Republic of Congo; 5 Laboratory of Clinical Microbiology, University of Liège, 4000 Liège, Belgium; 6 Department of Internal Medicine, Faculty of Medicine, University of Kinshasa, Kinshasa, Democratic Republic of Congo; 7 National Malaria Control Programme, Kinshasa, Democratic Republic of Congo

**Keywords:** Molecular, Markers, Anti-malarial, Resistance, Treatment, Failure, Democratic Republic of Congo

## Abstract

**Background:**

The national policy for malaria treatment of the Democratic Republic of Congo recommends two first-line artemisinin-based combinations for the treatment of uncomplicated malaria: artesunate-amodiaquine and artemether-lumefantrine. This study investigated the presence of markers associated with resistance to the current first-line artemisinin-based combination therapy (ACT) in isolates of *Plasmodium falciparum* from treatment failure patients in the Democratic Republic of Congo.

**Methods:**

From November 2018 to November 2019, dried blood spots were taken from patients returning to health centres for fever within 28 days after an initial malaria treatment in six sentinel sites of the National Malaria Control Programme across Democratic Republic of Congo. The new episode of malaria was first detected by a rapid diagnostic test and then confirmed by a real-time PCR assay to define treatment failure. Fragments of interest in *pfk13* and *pfcrt* genes were amplified by conventional PCR before sequencing and the *Pfmdr1* gene copy number was determined by a TaqMan real-time PCR assay.

**Results:**

Out of 474 enrolled patients, 364 (76.8%) were confirmed positive by PCR for a new episode of *P. falciparum* malaria, thus considered as treatment failure. Of the 325 *P. falciparum* isolates obtained from 364 *P. falciparum*-positive patients and successfully sequenced in the *pfk13-*propeller gene, 7 (2.2%) isolates carried non-synonymous mutations, among which 3 have been previously reported (N498I, N554K and A557S) and 4 had not yet been reported (F506L, E507V, D516E and G538S). Of the 335 isolates successfully sequenced in the *pfcrt* gene, 139 (41.5%) harboured the K76T mutation known to be associated with chloroquine resistance. The SVMNT haplotype associated with resistance to amodiaquine was not found. None of the isolates carried an increased copy number of the *pfmdr1* gene among the 322 *P. falciparum* isolates successfully analysed.

**Conclusion:**

No molecular markers currently known to be associated with resistance to the first-line ACT in use were detected in isolates of *P. falciparum* from treatment failure patients. Regular monitoring through in vivo drug efficacy and molecular studies must continue to ensure the effectiveness of malaria treatment in Democratic Republic of Congo.

## Background

*Plasmodium falciparum* is the most widespread *Plasmodium* species and is responsible for most severe forms of malaria, and death related to malaria in sub-Saharan Africa. *Plasmodium falciparum* has developed resistance mechanisms against almost all existing anti-malarial drugs, and this is a major threat to malaria control worldwide. The high level of *P. falciparum* resistance to chloroquine (CQ) and then to sulfadoxine-pyrimethamine (SP) has led the Democratic Republic of Congo (DRC), like all endemic countries, to change its anti-malarial drug policy for the treatment of uncomplicated malaria. The DRC national policy currently supports two first-line artemisinin-based combinations for the treatment of uncomplicated *P. falciparum* malaria: artemether–lumefantrine (AL) and artesunate–amodiaquine (ASAQ). If one of the first-line treatments is not available or is poorly tolerated by the patient, the other can be used. In case of treatment failure (confirmed by microscopy) with both first-line artemisinin-based combinations, the patient should be given a dual therapy of quinine plus clindamycin or plus doxycycline, while dihydroartemisinin-piperaquine (DP) could be used in a one-time episode [1].

The World Health Organization (WHO) recommends regular surveillance of artemisinin-based combination therapy (ACT) efficacy to provide an early warning against the emergence and spread of resistance [2]. Numerous polymorphisms in the *P. falciparum* genome have been suggested to provide resistance to ACT, both to artemisinin and the associated drugs [3, 4]. Mutations in the propeller domain of the *P. falciparum kelch 13* gene (*pfk13*) have been associated with in vivo delayed parasite clearance and in vitro artemisinin (ART) resistance in the ring stage survival assay [5, 6]. These mutations have spread within the Greater Mekong Subregion of Southeast Asia and have been classified as validated markers and candidate/associated markers of ART resistance [7]. Some of these mutations are increasingly being detected in sub-Saharan African countries, providing evidence for de novo emergence of resistance to artemisinin in Africa [8–11]. Mutations in the chloroquine resistance transporter gene (*pfcrt*) originally identified as a marker of CQ resistance, have also been associated with resistance to amodiaquine (AQ) [12]. The *pfcrt* gene is highly polymorphic in codon position 72–76, determining different haplotypes that include the key mutation K76T associated with CQ resistance, while the SVMNT haplotype has been associated with AQ resistance [13]. Additionally, an increased copy number of the gene encoding the multidrug resistance 1 transporter (*pfmdr1*) has been postulated to confer resistance to lumefantrine (LU) [14]. With the currently recommended artemisinin-based combinations, the absence of resolution of fever and parasitaemia or their recurrence within 28 days of treatment is considered as treatment failure, while all recurrence of fever and parasitaemia after 28 days of an initial treatment should, from an operational standpoint, be considered as re-infection and treated with first-line ACT [15]. The distinction between recrudescence and reinfection may be confirmed only by genotyping of parasites from initial and recurrent infections. This concern is addressed by therapeutic efficacy studies according to the World Health Organization (WHO) protocol [16]. Treatment failure is the inability to clear parasites from a patient’s blood or to prevent their recrudescence after the administration of an anti-malarial drug. Many factors can contribute to treatment failure, including resistance and inadequate exposure to drug due to sub-optimal dosing, poor patient compliance, poor drug quality, vomiting, poor drug absorption and drug interactions. The present study investigated the presence of molecular markers in *pfcrt*, *pfk13* and *pfmdr1* genes associated with resistance to current first-line ACT in *P. falciparum* from treatment failure patients in the DRC.

## Methods

### Study area

Six sentinel sites of the National Malaria Control Programme (NMCP) were selected among 26 provinces of the DRC. The NMCP sentinel sites are organized within one selected Health Zone entity, per province, and are part of a national network for malaria surveillance. The study sites included 2 of the 3 largest cities of the country and 4 other sites were selected based on their epidemiological facies and their accessibility. Thus, the following sentinel sites were selected: for the largest cities, Kingasani in Kinshasa, the capital city of the DRC and Kabondo in Kisangani; for the equatorial facies, Bolenge in Equateur province and Vanga in Kwilu province; for the tropical facies, Fungurume in Lualaba province; and for the mountainous facies, Katana in Sud-Kivu province. In each study site, 1 to 3 health centres were selected based on their accessibility and attendance for performing the study.

### Study patients

From November 2018 to November 2019, patients of all ages were seen on day 0 for the initial malaria episode and, in case of the absence of resolution or the recurrence of fever within 28 days of the artemisinin-based combination treatment, the patients were encouraged to return to the health centre for follow up. Returning patients who had a positive malaria rapid diagnostic test (RDT) were enrolled after receiving informed consent. The new malaria episode in returning patients was afterward confirmed by a *P. falciparum* real-time PCR assay to define treatment failure. Patients who reported an incomplete ACT during the initial episode were excluded from the study.

### Blood sample collection

Screening tests were performed on blood samples taken by finger prick. Malaria was detected using RDTs available on site: SD Bioline Malaria Ag P.f (Standard Diagnostics, South Korea) or CareStart Malaria Pf (Access Bio, South Korea). After enrollment, a blood sample was taken by finger prick and three spots were deposited on Whatman Grade GB003 filter paper (Whatman, GE Healthcare). Dried blood spots (DBS) were placed in an individual grip seal plastic bag containing silica gel desiccant and were then stored at – 20 °C before molecular analysis.

### DNA extraction

DNA was extracted from blood spots using the QIAamp DNA Mini Kit (Qiagen, Germany) following the manufacturer’s recommended protocol for DBS. The extracted DNA was stored at – 20 °C before PCR testing.

### Detection of *P. falciparum* by real-time PCR

A real-time PCR for the detection of *P. falciparum* was performed according to a modified version of a previously described procedure [17]. Briefly, the mixture contained: 1 × Taqman Universal PCR Master Mix (Applied Biosystems, USA), 200 nM *P. falciparum* primers and probe, 15 µM primers and 5 µM probe of the host β-globin as internal positive control and 5 µl of DNA template in a total volume of 25 µl. Assays were run on an ABI 7500 Fast real-time thermocycler (Applied Biosystems, USA) at the Laboratory of Molecular Biology of the School of Medicine, University of Kinshasa, DRC.

### pfk13 and pfcrt genes polymorphisms

The target sequence was a 506-nucleotide fragment of the *pfk13*-propeller gene covering codon-positions 427–595, following a procedure previously described [18]. This segment of interest includes mutations associated with ART resistance [7]. For the *pfcrt* gene, the 152-nucleotide fragment of interest (containing codon-positions 72–76) was amplified following a previously described procedure [19]. PCR assays were run using a conventional thermal cycler Hybaid HBPXE 0.2 (Thermo Scientific, USA) at the Laboratory of Molecular Biology of the School of Medicine, University of Kinshasa, DRC. After purification using AMPure XP magnetic beads (Beckman Coulter, USA), the PCR products were added to a mix of Big Dye Terminator V3.1 for the sequencing reaction. The resulting nucleotide sequences were analysed on an ABI 3730 DNA Analyzer automated sequencer (Applied Biosystems, USA) using the Sanger method at GIGA (University of Liège’s Interdisciplinary Research Institute in the Biomedical Sciences). Sequences (forward and reverse) were aligned using Vector NTI (Thermo Fisher Scientific, US) and compared to the reference sequence PF3D7_0709000 (https://www.ncbi.nlm.nih.gov/gene/term=PF3D7_0709000, accessed March 2020) for *pfcrt* and PF3D7_1343700 (http://www.plasmodb.org, accessed March 2020) for *pfk13* using the online Basic Local Alignment Search Tool (BLAST) [National Center for Biotechnology Information (NCBI), USA] for identifying mutations.

### Estimation of *pfmdr1* copy number

The *Pfmdr1* copy number was assessed by a relative quantification real-time PCR using an ABI 7500 thermocycler (Applied Biosystems, US), as previously described [20]. In each run of real-time PCR, 3D7 and Dd2 clones were used as calibration controls with single and multiple copies of the *pfmdr1* gene, respectively 1 copy and 4 copies, and a negative control containing no template DNA was also included. Test samples were assayed in triplicate, the copy number of *pfmdr1* was determined using the comparative ΔΔCt method and calculated as 2^–∆∆Ct^. Samples with a spread of ∆∆Ct among the three triplicates of more than 1.5 or with a Ct > 35 were repeated and the second result used. Analyses were performed at the Laboratory of Clinical Microbiology of the University of Liege, Belgium.

### Statistical analysis

Data were entered in an Excel 2010 spreadsheet by an independent data clerk. Statistical analysis was performed using SPSS V. 20.0 (IBM Corp., Armonk, USA). Samples for which the genotype could not be determined were excluded from the analysis. Categorical variables were expressed as frequencies with 95% confidence intervals (95% CI) while quantitative variables were expressed as median with interquartile range (IQR). The mutant and wild-type alleles identified in the sequenced isolates were used to generate the prevalence of mutations. Proportions in different characteristics of patients were compared by Chi-square. Tests were two-sided, *p*-values < 0.05 were considered significant.

## Results

### Baseline characteristics of patients

In total, 474 patients were enrolled in the study, their age ranged from 0 to 76 years with a median age of 10 years (IQR: 4–21 years). The male to female sex ratio was 0.74. As shown in Table 1, the distribution of patients per age range and per study site was heterogeneous (*p* value < 0.001) and more than half of them were female (*p* value < 0.001). The treatment of the initial episode of malaria was based on AL, ASAQ and DP, respectively in 273 (57.6%), 196 (41.3%) and 5 (1.1%) patients. DP was used only in Kingasani (Kinshasa) while both AL and ASAQ were used in all sites, except in Bolenge where only AL was used during the study. Real-time PCR analysis of DNA extracted from DBS samples confirmed the new episode of *P. falciparum* infection for 364 (76.8%; 95% CI 72.7–80.5%) patients, considered treatment failure patients.

**Table 1:** Baseline characteristics of enrolled patients

Characteristics	*N*	% (95% CI)	*p*
Age (years)			< 0.001
0–5	172	36.3 (32.0–40.8)	
6–14	124	26.2 (22.3–30.4)	
15–49	154	32.5 (28.3–36.9)	
50–76	24	5.1 (3.3–7.5)	
Total	474	100.0	
Sex			< 0.001
Female	272	57.4 (52.8–61.9)	
Male	202	42.6 (38.1–47.2)	
Total	474	100.0	
Anti-malarial used for initial episode			0.009
ASAQ	196	41.4 (36.9–45.9)	
AL	273	57.6 (53.0–62.1)	
DP	5	1.1 (0.3–2.4)	
Total	474	100.0	
Enrolled patients per site			< 0.001
Bolenge	88	18.6 (15.2–22.4)	
Fungurume	83	17.5 (14.2–21.2)	
Kabondo	20	4.2 (2.6–6.4)	
Katana	143	30.2 (26.1–34.5)	
Kingasani	113	23.8 (20.1–27.9)	
Vanga	27	5.7 (3.8–8.2)	
Total	474	100.0	
Detection of *P. falciparum* by PCR			< 0.001
Negative	110	23.2 (19.5–27.3)	
Positive	364	76.8 (72.7–80.5)	
Total	474	100.0	

*AL* artemether-lumefantrine, *ASAQ* artesunate-amodiaquine, *DP* dihydroartemisinin-piperaquine, *N* number

### *Pfcrt* 72–76 haplotype for eventual resistance to amodiaquine

Of the 364 *P. falciparum* isolates detected by real-time PCR, 335 were successfully sequenced among which 139 (41.5%; 95% CI 36.2–47.0%) harboured the K76T mutation known to confer resistance to CQ. The CVIET haplotype was found in 98 (70.5%) isolates harbouring the K76T mutation, followed by CVIKT (20.1%), CVINT (5.8%) and CVMNT (3.6%). The SVMNT haplotype associated with resistance to AQ was not detected. Among isolates harbouring the K76T mutation, 65/133 (48.9%) of them were found in patients treated with ASAQ while 74/198 (37.4%) occurred in patients treated with AL (*p* value = 0.038).

### *Pfk13*-propeller polymorphism for eventual resistance to artemisinin

Of the 325 successfully sequenced *P. falciparum* isolates, 318 (97.8%; 95% CI 95.6–99.1%) were wild-type and 7 (2.2%; 95% CI 0.9–4.4%) carried non-synonymous (NS) mutations in the *pfk13*-propeller domain. Among these, 3 have been previously described (N498I, N554K and A557S) and 4 were newly reported (F506L, E507V, D516E and G538S). None of the mutations that have been validated or suspected to be associated with ART resistance were found in the study.

The N498I, D516E and G538S mutations were each found in isolates also harbouring the K76T mutation. Table 2 shows the distribution of mutations in the *pfk13*-propeller gene and the key mutation K76T in the *pfcrt* gene among enrolled treatment failure patients per site.

**Table 2:** Distribution of *pfk13* and *pfcrt*-K76T mutations

Site	Positive PfPCR N (%)	*Pfcrt* gene			*pfK13* gene			
		Pf successfully sequenced N	K76T N	K76T % (95% CI)	Pf successfully sequenced N	Mutant Pf N	Mutant Pf % (95% CI)	NS mutation
Bolenge	70 (79.5)	62	21	33.9 (22.3–47.0)	60	2	3.3 (0.4–11.5)	F506L, N554K
Fungurume	63 (75.9)	60	6	10.0 (3.8–20.5)	58	1	1.7 (0.0–9.2)	A557S
Kabondo	17 (85.0)	16	3	18.8 (4.0–45.6)	17	0	0.0 (0.0–16.2)	–
Katana	95 (66.4)	91	70	76.9 (66.9–85.1)	86	2	2.3 (0.3–8.1)	N498I, D516E
Kingasani	92 (81.4)	81	27	33.3 (23.2–44.7)	80	2	2.5 (0.3–8.7)	E507V, G538S
Vanga	27 (100.0)	25	12	48.0 (27.8–68.7)	24	0	0.0 (0.0–11.7)	–
Total	364 (76.8)	335	139	41.5 (36.2–47.0)	325	7	2.2 (0.9–4.4)	

*N* number, *PfPCR* *Plasmodium falciparum* polymerase chain reaction, *NS* non-synonymous

### *Pfmdr1* copy number for eventual resistance to lumefantrine

In total, 322 *P. falciparum* isolates from enrolled treatment failure patients were successfully analysed for copy number variation in the *pfmdr1* gene. Using a copy number threshold of 1.5 to define multiple copies, all isolates harboured a single copy of the *pfmdr1* gene.

## Discussion

The present study has not detected molecular markers associated with resistance to first-line ACT in use in the DRC in treatment failure patients. Although none of the mutations associated with ART resistance in Southeast Asia have been found in this study, 7 coding substitutions that are of unknown phenotype were observed, among which 3 have been previously reported, notably N498I in Kenya [21], N554K in Comoros and A557S in Togo and DRC [22], and 4 others that had not yet been reported (F506L, E507V, D516E, G538S). Numerous *pfk13*-propeller mutations of unknown function are commonly reported in sub-Saharan African countries, including the DRC [18, 22, 23]. There are criteria for prioritizing further laboratory studies, notably the frequent observation of a new allele with a non-synonymous mutation, the evidence of dissemination and preliminary association with clinical data whenever possible [23]. Several independent single nucleotide polymorphisms (SNPs) could be responsible for the ART resistance in sub-Saharan Africa, as the known mutations that confer drug resistance would differ from one location to another, depending on the parasite genetics. There is the possibility that *pfk13* mutations do not cause ART resistance in isolation, but could act in combination with other genetic or non-genetic factors that are different in African and Southeast Asian parasite populations [24, 25]. Since African *pfk13*-propeller mutations were shown to be different from those found in Southeast Asia, further molecular and biochemical studies should investigate whether other factors such as additional mutations could be associated with altered functions of the PFK13 protein, resulting in altered ART sensitivity. However, some of the validated and candidate mutations associated with ART resistance in Southeast Asia have been detected in the DRC [11, 26] and in some neighbouring countries, such as Rwanda [8, 9] and Uganda [10], providing evidence of de novo emergence of ART resistance in sub-Saharan Africa. Thus, surveillance must be strengthened to avoid the worst.

The global prevalence of the K76T mutation known to be associated with CQ resistance was 41.5%, but this was variable from one site to another, ranging from 10.0% in Fungurume to 76.9% in Katana. In 2017, a study conducted in 10 sites (including 6 sites of the present study) reported a global prevalence of the K76T mutation of 28.5% but with a high between-regions variability ranging from 1.5% in Fungurume to 89.5% in Katana [27]. In the present study, the prevalence of K76T in patients treated with ASAQ (48.9%) was higher than in those treated with AL (37.4%) with low statistical difference (*p* value = 0.038). The possibility that AQ could continue to contribute to selection for the K76T mutant even after discontinuance of CQ usage has been previously raised [28]. The simultaneous presence of very low and high prevalence of CQ resistance could be related to a between-regions difference of CQ pressure and also to the effect of selection for CQ resistance depending on the genetic structure of parasite populations, which have been shown to vary significantly across the country [29]. Data concerning current CQ use in the country are not available, further studies at the community level and parasite genetic studies should be conducted to explain the persistence of a high CQ resistance rate in some provinces despite the withdrawal of this molecule from the national policy of malaria treatment. The SVMNT haplotype associated with AQ resistance was not detected in the present study, which is encouraging for the DRC national policy for the continued use of AQ in ACT. This haplotype has not yet been reported in the DRC [19, 26, 27, 30] whereas it was found in neighbouring countries, such as Tanzania and Angola [31, 32].

None of the *P. falciparum* isolates had multiple copies of the *pfmdr1* gene in this study, consistent with the general absence [33, 34] or low frequencies [26, 35, 36] of *pfmdr1* copy number variation in *P. falciparum* from sub-Saharan Africa. The multiple copy number of the *pfmdr1* gene has been postulated to confer resistance to lumefantrine [14], the drug associated to AL in one of the artemisinin-based combinations used in the DRC.

The artemisinin-based combinations used to treat the initial episode were those which were available on each study site during the investigation and best tolerated by the patient. In practice, AL tends to be primarily used in urban areas because patients have more options to obtain it from private pharmacies, while ASAQ is used in rural areas. The use of DP as a first-line ACT in five patients in Kinshasa constituted a case of non-adherence to treatment guidelines often observed in urban areas [1]. The study assessed molecular markers of anti-malarial resistance in treatment failure patients. Many other factors not assessed by the present study can contribute to treatment failure, notably those related to poor exposure to drugs. The non-homogeneous distribution of enrolled treatment failure patients across different characteristics suggests that some of these factors may be dependent on age, gender and region. When possible, treatment failure must be confirmed by microscopy, as histidine rich protein-2 (HRP-2)-based RDT may remain positive for weeks after successful treatment due to persistent antigenaemia, even without recrudescence [37, 38]. In this study, a *P. falciparum* real-time PCR assay was used afterwards to confirm *P. falciparum* malaria in patients returning to health centres for fever within 28 days of an initial malaria treatment.

The study contributes to the ongoing surveillance of resistance to anti-malarial drugs in use in the DRC as recommended to track the emergence and spread of *P. falciparum* resistance to different molecules used in malaria management. However, although the study was carried out in six provinces of the DRC with varied geography and malaria endemicity, the results were not representative of either any one province or the entire country.

## Conclusion

No molecular marker currently known to be associated with resistance to first-line ACT components in use in the DRC was detected in *P. falciparum* isolated from treatment failure patients. The findings are supportive of the DRC’s current malaria treatment policy, however, the appearance of new mutants with as-yet unknown functions calls for further investigation. In addition, the other factors, apart from the drug resistance, that could contribute to treatment failure must be assessed in order to ensure the efficacy of the treatment of malaria in the DRC.

## Supplementary Information

**Additional file 1**: *Pfk13* and *pfcrt* sequences for wild and mutant isolates.

**Additional file 2:**: Minimal data set containing raw data.

## Data Availability

All data generated and analysed during this study are included in this published article.

## Abbreviations

ACT: Artemisinin-based combination therapy
AL: Artemether-lumefantrine
AQ: Amodiaquine
ASAQ: Artesunate-amodiaquine
CQ: Chloroquine
DBS: Dried blood spot
DNA: Deoxyribonucleic acid
DP: Dihydroartemisinin-piperaquine
DRC: Democratic Republic of Congo
IQR: Interquartile range
LU: Lumefantrine
NMCP: National Malaria Control Programme
PCR: Polymerase chain reaction
*pfcrt*: *Plasmodium falciparum* Chloroquine resistance transporter
*pfk13*: *Plasmodium falciparum* Kelch 13
*pfmdr1*: *Plasmodium falciparum* Multidrug resistance1
RDT: Rapid diagnostic test
SNP: Single nucleotide polymorphisms
SP: Sulfadoxine-pyrimethamine
WHO: World Health Organization
